# Supplementary material for: MNase Digestion Protection Patterns of the Linker DNA in Chromatosomes
Source: Cells. 2021 Aug 29;10(9):2239. doi: 10.3390/cells10092239 (PMC8469290; doi:10.3390/cells10092239)
Supplement: Supplementary file 1 [file cells-10-02239-s001.zip › cells-1355685-supplementary.pdf]

Supplementary Material and Methods

Preparation of single-stranded DNA

E.coli DH11S (F+) cells carrying the recombinant pBluescript-globin plasmids were infected with M13KO7 helper phage to prepare the single-stranded DNA. 100 ml TBG was inoculated with 300 μl DH11S SOB plasmid glycerol and 500 μl of M13KO7 helper phage (~ 10^{11} pfu/ml), and incubated at 37 ^{o}C. After 90 minutes incubation, 100 μg/ml of ampicillin and 70 μg/ml of kanamycine were added, and the culture grown for a further 12 to 14 hours.

Phage particles were isolated by spinning the culture twice at 8K for 20 minutes at 4°C. The 2° supernatant was added to 0.25 volume of 40 % PEG6000 in 2.5 M NaCl and incubated for 60 minutes on ice to precipitate the phage particles. The phage particle was collected by spinning at 8K for 30 minutes at 4 ^{o}C. The pellet was further spun at 3K for 5 minutes at 4°C and the residual PEG/NaCl was removed completely. The pellet was resuspended in 400 μl TE and split into two 200 μl aliquots. 1 ml of Tri Reagent (Sigma) was added to each aliquot and the mixture incubated at room temperature for 15 minutes with occasional vortex, 200 μl of chloroform was added and the mixture was incubated at room temperature for a further15 minutes with occasional vortex. After this incubation, the mixture was spun at 13 K for 15 minutes and the colourless aqueous phase removed (containing RNA). The remaining interphase and organic phase contained single-stranded DNA and protein. To precipitate the DNA, 0.3 ml of ethanol was added and the mixture incubated at room temperature for 15 minutes. The DNA pellet was collected by spinning at 6.5K for 5 minutes, all residual supernatant removed, and the pellet washed twice with 1 ml of 0.1 M sodium citrate/10 % ethanol, then twice with 70 % ethanol. The dried pellet was resuspended

in 675 μl TE and then EDTA added to 2.5 mM, SDS added to 0.5%, and NaAc pH 5.2 added to 0.3 M followed by extraction with three to four times phenol and once with chloroform. After ethanol precipitation, the DNA was resuspended in 500 μl TE and incubated on ice for 60 minutes with 300 μl 20 % PEG6000 in 2.5 M NaCl. After this, pellet was collected by spinning the mixture at 13K for 15 minutes followed by two washes, with 70 % ethanol and resuspension in 210 μl TE. The quality of single-stranded DNA was assessed by running two 5 μl samples, one incubated at room temperature and the other at 95 ^{o}C for 4 minutes, on a 1.6 % agarose gel.

Preparation of core histones, linker histones H1/H5, and recombinant GH1/GH5

Core histones from linker histone-depleted chicken erythrocyte chromatin were prepared by chromatography on hydroxyapatite as described previously (Peterson and Hansen, 2008). Linker histones H1 and H5 were prepared from chicken reticulocyte chromatin as described previously (Allan *et al*., 1980). For the preparation of recombinant GH1/GH5, the coding sequence for the globular domain of the chicken linker histone H1 and H5 were prepared by PCR and the purified amplification products were restricted with *Bam*HI/*Nde*I and cloned into the T7 polymerase-based expression vector pT79, a derivative of pMW172 (Way *et al*., 1990). The resulting constructs are pGH1, contained an in-frame H1 fragment encoding the 76 amino acid GH1 peptide with an additional N-terminal methionine residue, and pGH5, contained an in-frame H5 fragment encoding the 79 amino acid GH5 with an additional N-terminal methionine residue, respectively. After transformation, *E.coli* (*DE3*) containing pGH1 or pGH5 was grown in 200 ml LB and induced in mid-log phase by the addition of IPTG. After 3 hours induction, the culture was harvested and the pellet was collected by centrifugation at 5000 rpm for 10 min at 4°C. Subsequently, the pellet was resuspended in 25 ml grinding buffer (50 mM Tris-Cl pH 7.9,

2 mM EDTA, 0.1 mM DTT, 1mM β-mercaptoethanol, 0.5 M NaCl, 0.1 % sodium deoxycholate, 5 % glycerol and 25 μg/ml PMSF) with homogenization and sonication. The suspension was spun 16000 rpm for 15 minutes at 4°C. The supernatant was collected and 70% perchloric acid (PCA) was added to a final concentration of 5% (V/V). The PCA extraction was allowed to progress on ice for 1 hour whereupon the insoluble material was pelleted by spinning 16000 rpm for 30 minutes at 4°C. The supernatants were pooled and the protein precipitated by the addition of 6 volumes of acetone and incubation at –20°C overnight. The precipitate was collected by centrifugation and the pellets were washed 3 times in acetone and dried in vacuum. Fractionation of soluble GH1 and GH5 proteins was achieved by ion-exchange chromatography using the cation exchanger sephadex CMC-25 (Pharmacia). Briefly, the dried pellet of acid-soluble GH1 or GH5 protein was dissolved in 1.2 ml of H_2O containing 0.1 mM PMSF. 3 ml of CMC-25 was packed into a column and equilibrated with 10 ml of 20 mM phosphate. Subsequently, 1 ml of sample, containing 490 μl acid-soluble GH1 or GH5, 10 μl of 1 M phosphate pH 6.8, and 500 μl of 20 mM phosphate was loaded into the top of the column. The column was then washed with (i) 5 ml of 20 mM phosphate, (ii) 5 ml of wash buffer (20 mM phosphate, 200 mM NaCl), and (iii) 5 ml of elution buffer (20 mM phosphate, 700 mM NaCl). Fractions were collected and measured absorbance at 280 nm. Peak fractions containing GH1 or GH5 were pooled and stored at –20°C.

1. Allan J, Staynov DZ, Gould H. 1980. Reversible dissociation of linker histone from chromatin with preservation of internucleosomal repeat. *Proc Natl Acad Sci U S A.* 77, 885-889. PMID: 6928686

2. Peterson CL, Hansen JC. 2008. Chicken erythrocyte histone octamer preparation. *CSH Protoc*. 2008 Dec 1. PMID: 21356757.

3. Way M, Pope B, Gooch J, Hawkins M, Weeds AG. 1990. Identification of a region in segment 1 of gelsolin critical for actin binding. *EMBO J.* 9, 4103-4109. PMID: 2174356

Monomer extension reactions

A schematic outline of the monomer extension procedure has been shown in Figure 1C. A 20 to 50 ng sample of freshly denatured monomer DNA was mixed with 0.8 to 1.5 μg of the appropriate single-stranded DNA (according to the size of the single-stranded DNA) in 100 mM NaCl, 2 mM DTT, 20 mM Tris pH 7.5 and 20 mM $MgCl_2$ to a total volume of 25 μl; annealing was effected by denaturation at 95 ^oC for 3 minutes, the temperature dropped to 80 ^oC for 30 seconds, and then gradually lowered to 55 ^oC over 45 minutes. After annealing, the reaction mix was diluted to 50 μl by the addition of dNTPs (10 μM final concentration of each dATP, dCTP, dTTP and dGTP), BSA (100 μg/ml final concentration), 5 units of *E. coli* DNA polymerase (Klenow fragment; Amersham), 20 units of appropriate restriction enzyme and water, and incubated at 37 ^oC for 60 minutes followed by phenol:chlorofom and chloroform:isoamyl alcohol extractions and sodium acetate/ethanol precipitation. The product was washed with 70 % ethanol and then resuspend in 8 to 10 μl of sequence stop mix. Samples were heat denatured and then electrophoresed in 6 % denaturing polyacrylamide gels with size standards of long-geared C and T ^{35}S sequencing reactions of M13mp18 ssDNA (USB) and ^{32}P end-labelled *Hin*FI and *Dde*I digests of phage lambda.

List of coordinates (position of dyad axis) of all 38 selected nucleosomes and chromatosomes in Figure 3.

-952, -931, -829, -797, -787, -784, -769, -763, -750, -746, -737, -730, -721, -696, -689, -660, -626, -560, -404, -365, -336, -320, -306, -291, -284, -255, -235, -224, -173, -163, -140, -130, -65, -51, -39, -2, +13, +22.

The coding sequence of recombinant GH1 used in this study.

ATG CCA GCA GGT CCA AGC GTC ACC GAG CTG ATC ACC AAG GCC GTG TCC
GCC TCC AAG GAG CGC AAG GGG CTC TCC CTC GCC GCG CTC AAG AAG GCG
CTT GCC GCC CGC GGC TAC GAC GTG GAG AAG AAC AAC AGC CGC ATC AAG
CTG GGG CTC AAG AGC CTC GTC AGC AAG GGC ACC CTG GTG CAG ACC AAG
GGC ACC GGC GCC TCG GGC TCT TTC AAG CTG AAT AAA AAG

The coding sequence of recombinant GH5 used in this study.

ATG TCG GCA TCG CAC CCC ACC TAC TCG GAG ATG ATC GCG GCG GCC ATC CGT
GCG GAA AAG AGC CGC GGC GGC TCC TCG CGG CAG TCC ATC CAG AAG TAC
ATC AAG AGC CAG TAC AAG GTG GGC CAC AAC GCC GAT CTG CAG ATC AAG
CTC TCC ATC CGA CGT CTC CTG GCT GCC GGC GTC CTC AAG CAG ACC AAA GGG
GTC GGG GCC TCC GGC TCC TTC CGC TTG GCC AAG AGC GAC AAG TGG

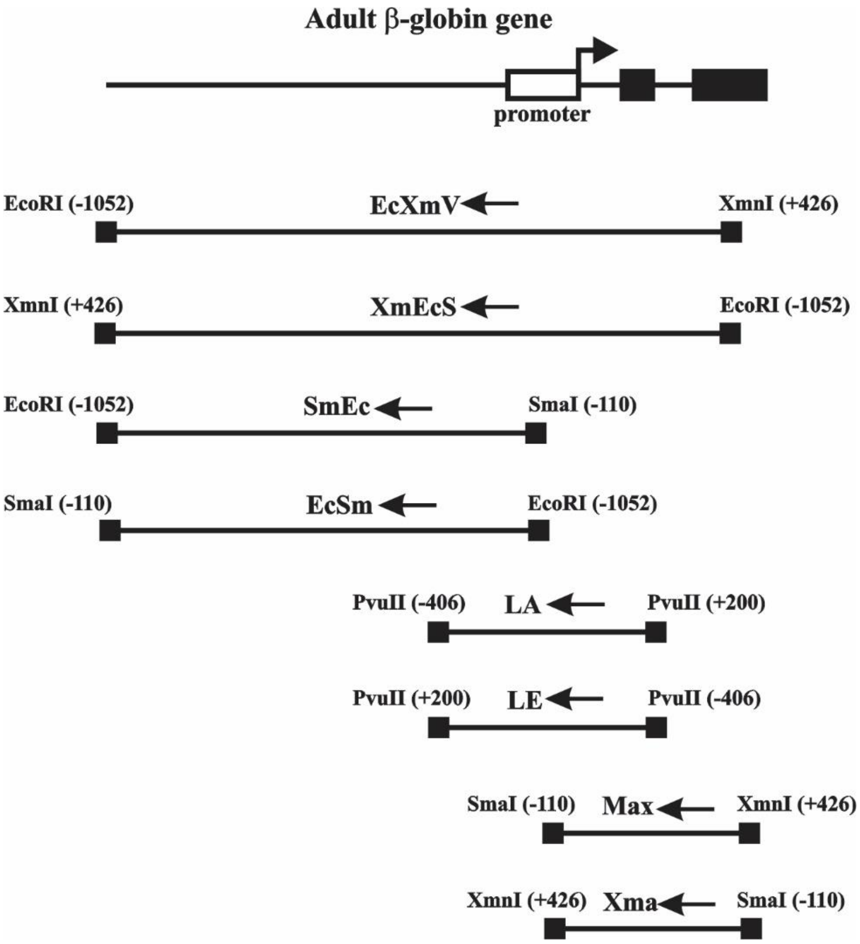

Figure S1

The location and orientation of the first 1.5 kb of the adult β-globin sequences contained within the mapping constructs employed in this study. The arrows on the mapping constructs indicate the direction of monomer extension on single-stranded DNA of each subclone.

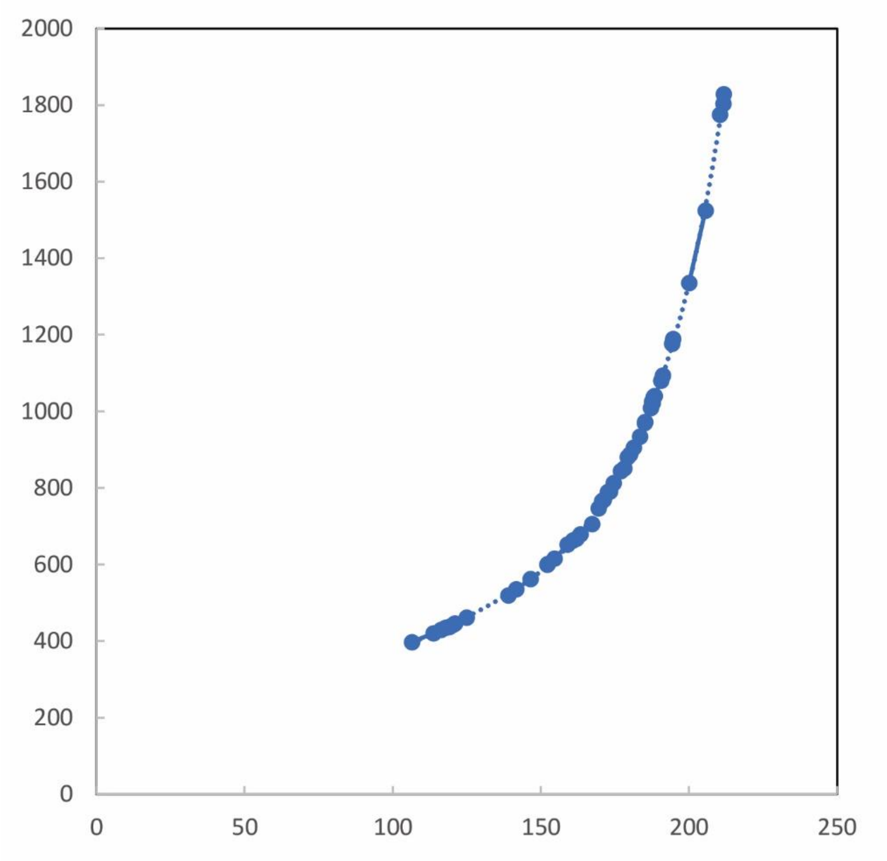

Figure S2

An example of correlation between DNA size and DNA mobility derived from analysis of restriction and sequencing markers on a 6% denaturing polyacrylamide gel. ($y = 0.0000000052631138441x^6 - 0.0000047994449096300x^5 + 0.0018196278760815800x^4 - 0.3657268145432680000x^3 + 41.0317370635442000000x^2 - 2{,}431.1887535852400000000x + 59{,}695.1162059215000000000$; $R^2 = 0.9997428796328580000$)

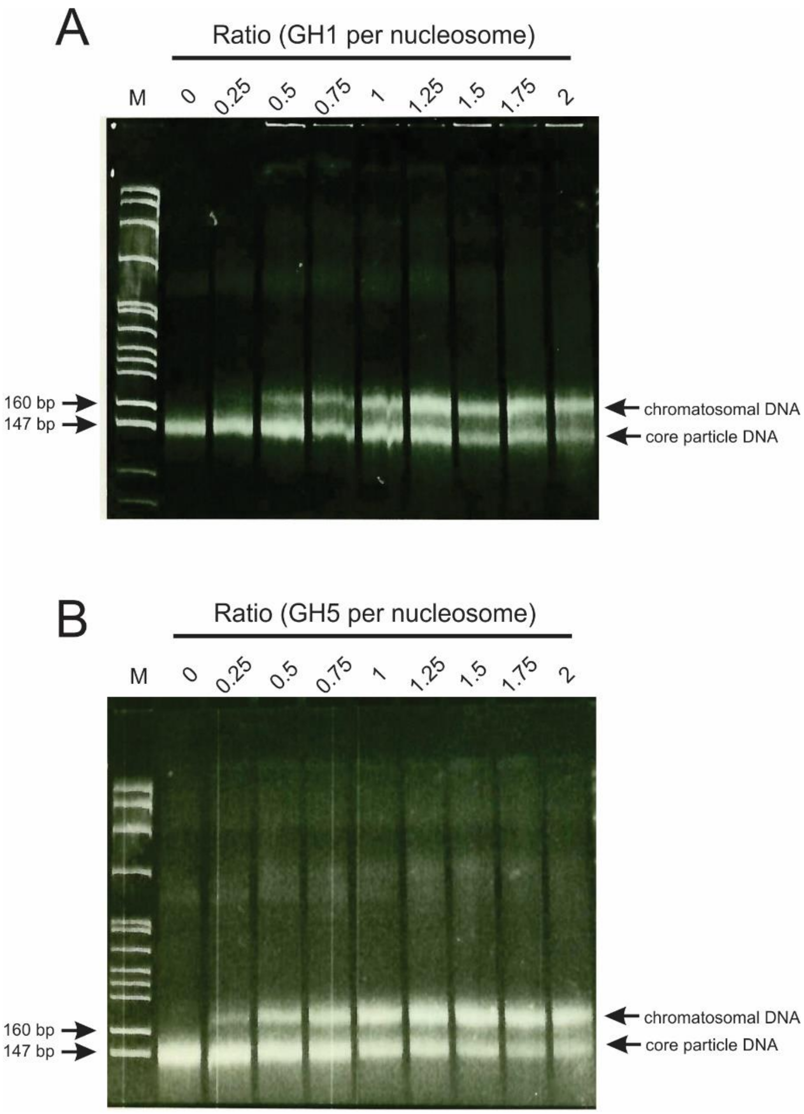

Figure S3

Chromatosome protection of reconstituted chromatin containing increasing amounts of (A) GH1 and (B) GH5. DNA purified from MNase digested chromatin were run in a 6% polyacrylamide gel. The linker histone globular domains to core histone octamer ratio is indicated above each lane. The marker (M) was an *Msp*I digest of pBR322 DNA.

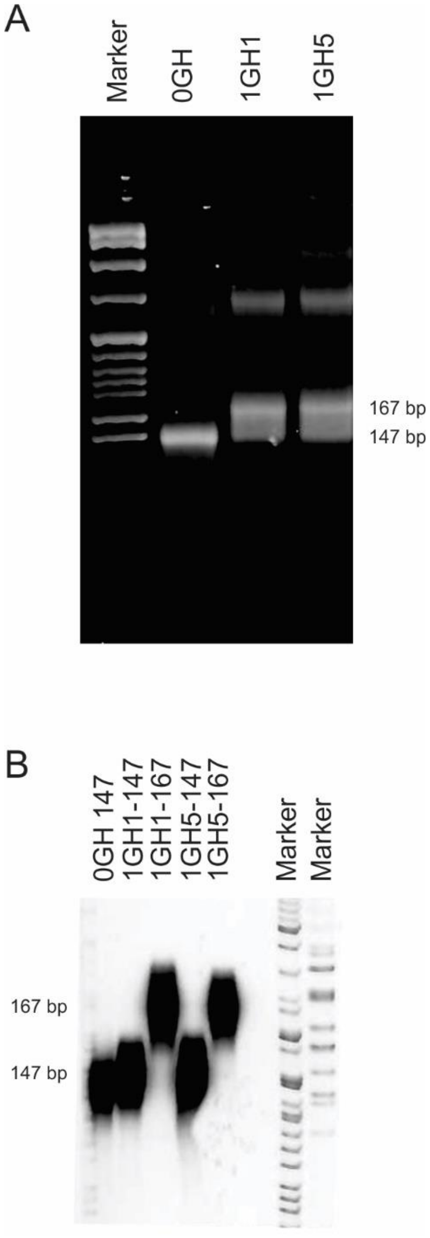

Figure S4

Gel analysis of core particle and chromatosomal DNAs. (A) A 4.5% metaphor agarose gel analysis of core and chromatosomal DNAs isolated from 37°C reconstitutes. (B) A 6% denaturing polyacrylamide gel analysis of 5’-end labelled core particle and chromatosomal DNAs.

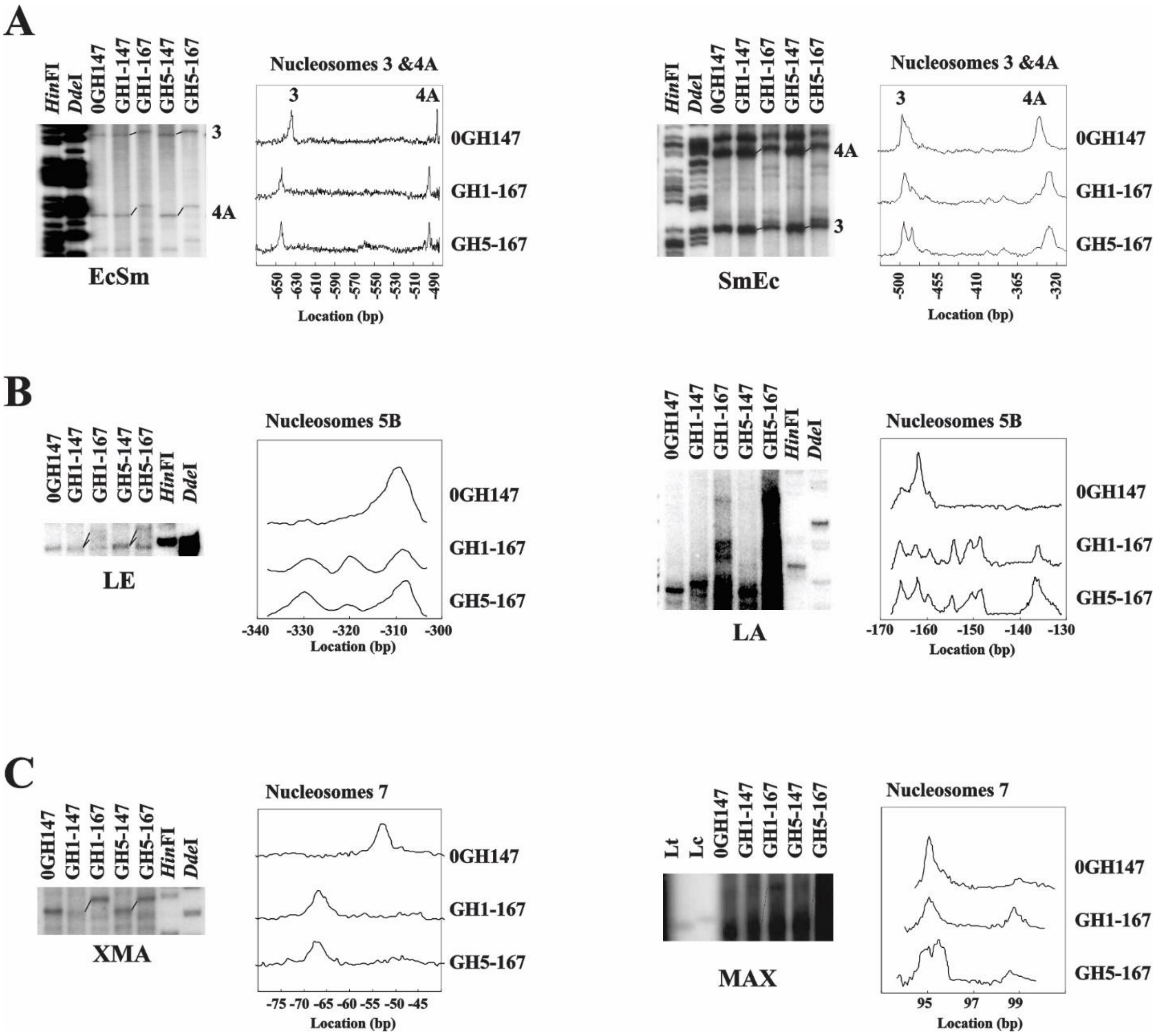
A
HinFI
DdeI
0GH147
GH1-147
GH1-167
GH5-147
GH5-167
3
4A
EcSm
Nucleosomes 3 &4A
0GH147
GH1-167
GH5-167
Location (bp)
SmEc
B
LE
Nucleosomes 5B
LA
C
XMA
Nucleosomes 7
Lt
Lc
MAX

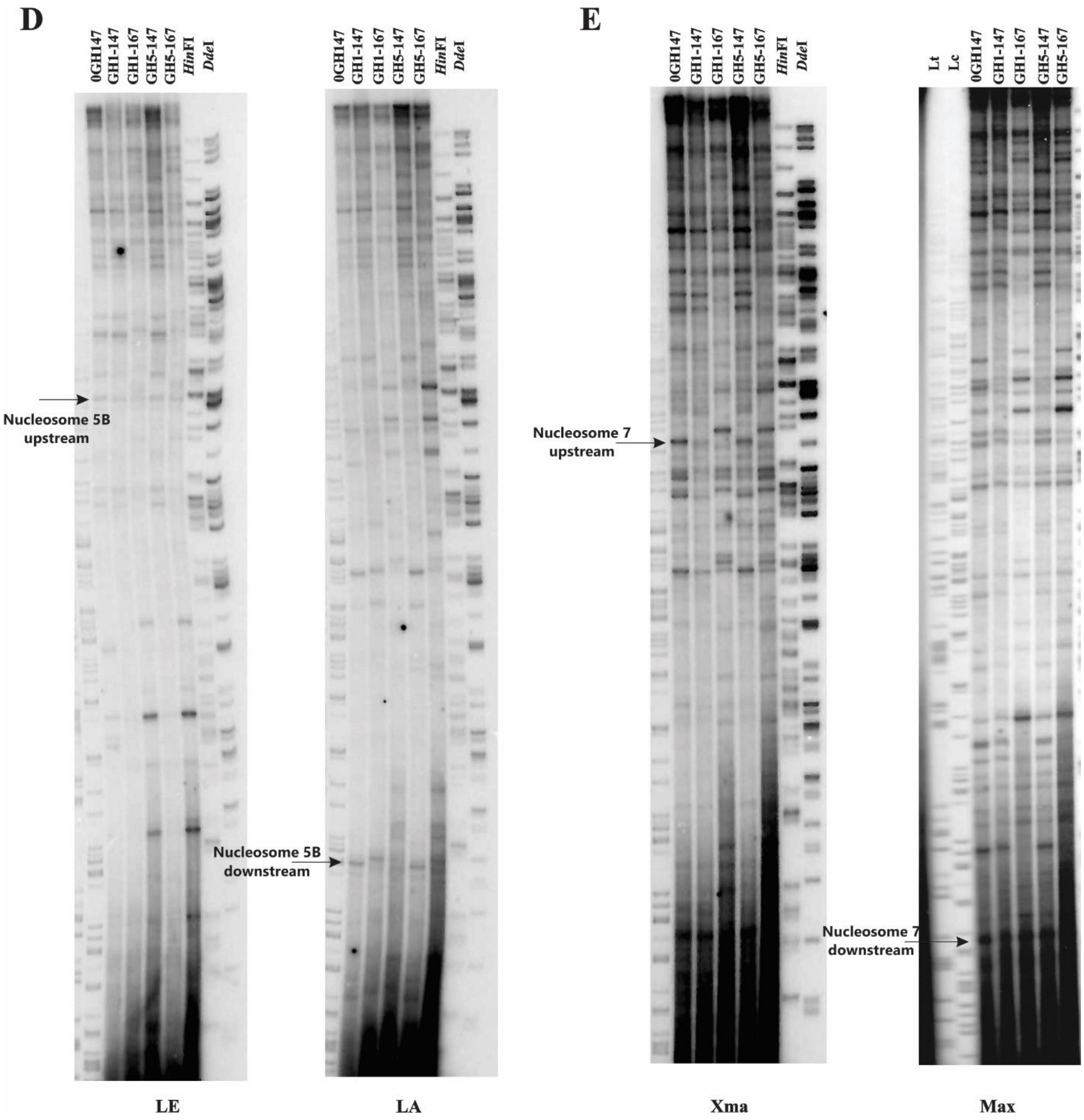

Figure S5

Distribution of lengths of extra DNA associated with chromatosomes compared to core particles. (A) A 6 % denaturing polyacrylamide gel analysis of nucleosomes 3 & 4A formed on mapping constructs EcSm and SmEc, and their densitometry analysis. (B) A 6 % denaturing polyacrylamide gel analysis of nucleosome 5B formed on mapping constructs LE and LA, and their densitometry analysis. (C) A 6 % denaturing polyacrylamide gel analysis of nucleosome 7 formed on mapping constructs MAX and XMA, and their densitometry analysis. Location is with respect the β-globin gene cap site. (D) A 6 % denaturing polyacrylamide gel analysis of

nucleosome 5B formed on mapping constructs LE and LA. (E) A 6 % denaturing polyacrylamide gel analysis of nucleosome 7 formed on mapping constructs Xma and Max.

Panel A: Nucleosomes 3 and 4A are well defined in both the upstream and downstream analyses. In Figure S5A, the left panel (Figure 2A, lanes 10-14) shows the upstream boundaries of core histone octamer positioning sites 3 and 4A map at –633 and -477 (Location is with respect to the β–globin cap site), and the right panel (Figure 2B, lanes 3-7) shows the downstream boundaries of core histone octamer positioning sites 3 and 4A map at –487 and -331. Core particle positioning sites derived from reconstitutes containing GH1 or GH5 have the same upstream and downstream coordinates as the core particle positioning sites derived from reconstitutes lacking linker histone globular domains.

The chromatosome positioning at sites 3 and 4A is independent of the type of globular domain employed during reconstitution and the upstream boundaries determined by these analyses are located at –644 and –-490 (left panel), and the downstream boundaries map to –477 and -321 (right panel).

This analysis demonstrates that for site 3 an extra 11 bp was protected at the upstream boundary in the chromatosome whereas an extra 10 bp of DNA was protected at the downstream boundary. Similarly, for site 4A an extra 13 bp was protected at the upstream boundary and an extra 10 bp was protected at the downstream boundary. Thus, for nucleosomes 3 and 4A the DNA extension in the chromatosome appears to be approximately equally distributed at each end of the core particle.

Panel B: The data presented for the analysis of core histone octamer positioning site 5B are analysed in the same way as above. Relevant regions of the gel analysis are taken from Figure S5D. The upstream boundary of the core histone octamer positioning site maps at –-308 and the corresponding chromatosomes appear to have upstream boundaries located at -308, and -331 (left panel). The result is independent of the type of globular domain employed during reconstitution.

A similar analysis has been employed to determine downstream boundaries for the 5B core particle and chromatosome positioning sites. These were mapped to -162 bp (core) and to -162 - (chromatosome) (right panel). Again their location was independent of globular domain type.

This analysis demonstrates that for site 5B the extra DNA protected at the upstream boundary in a chromatosome appears to be 0, or 23 bp. For the downstream boundary, an extra 0 bp were protected in the chromatosome. Thus, for Nucleosome 5B the additional DNA in the chromatosome appears to be asymmetrically (23+0) at each end of the core particle.

Panel C: The similar analysis approach has been applied to site 7 again. Relevant regions of the gel analysis are taken from Figure S5E. The upstream boundary of the core histone octamer positioning site maps at -51 and the corresponding chromatosomes appear to have upstream boundaries located at -66 (left panel), the downstream boundary of the core histone octamer positioning site maps at 95 and the corresponding chromatosomes appear to have upstream boundaries located at 95 and 99 (right panel). These results are independent of the type of globular domain employed during reconstitution.

The analysis shows a 15 nt extension for the upstream chromatosome boundary at site 7 and 4 nt and 0 nt extensions at the downstream boundary. Thus, the additional DNA in the chromatosome appears to be asymmetrically distributed (15+4) at the ends of the core particle.

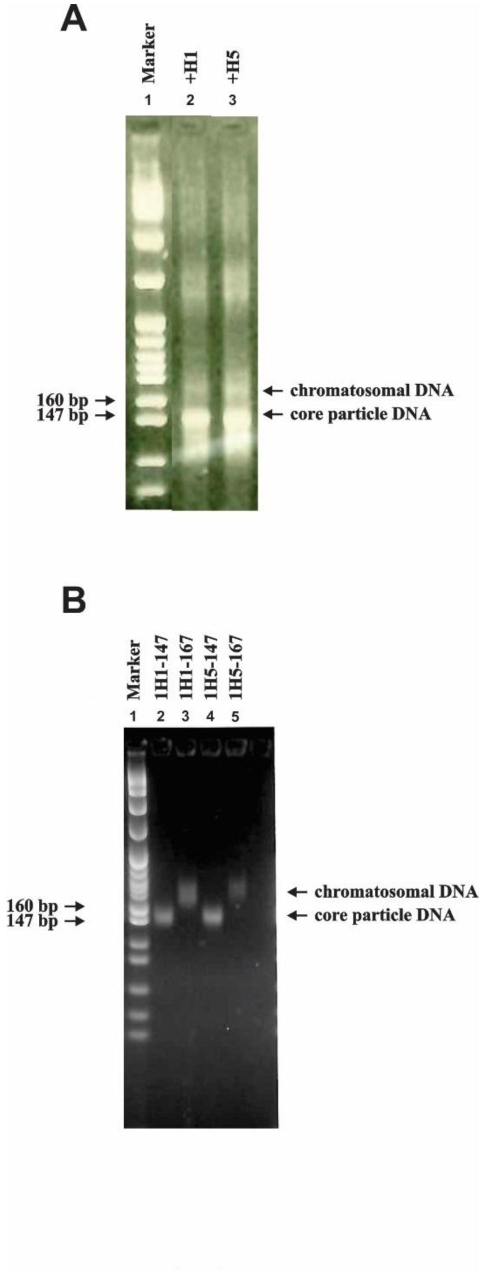

Figure S6.

(A) Chromatosome protection of reconstituted chromatin containing equal molar ration of recombinant H1 (+H1) or H5 (+H5). DNA purified from MNase digested chromatin were run in a 4.5% metaphor agarose gel. The marker was an *Msp*I digest of pBR322 DNA. (B) A 4.5% metaphor agarose gel analysis of core particle and chromatosomal DNAs. 1H1-147 & 1H5-147: core particle DNAs derived from H1- & H5-containing reconstitutes, respectively. 1H1-167 & 1H5-167: chromatosomal DNA derived from H1- & H5-containing reconstitutes, respectively.

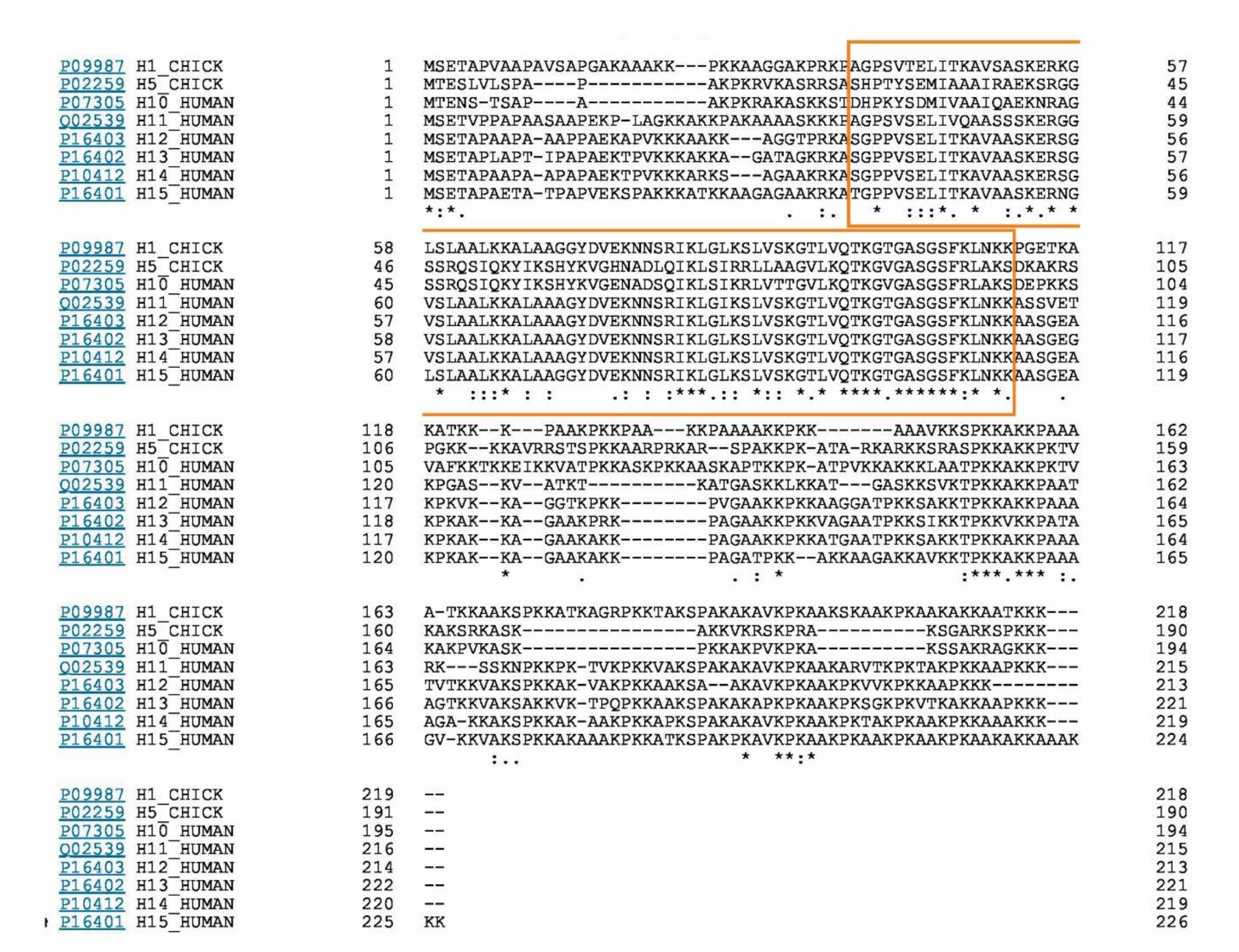

Figure S7.

Multiple sequence alignment of chicken linker histone variants (H1 and H5) and the somatic subtypes of human linker histones (H1.0, H1.1, H1.2, H1.3, H1.4, H1.5). The individual linker histone variants were aligned using CLUSTAL O(1.2.4). Box indicates globular domain region.
